# Dilatation of the Left Auricle

**Published:** 1901-06-08

**Authors:** 


### DILATATION OF THE LEFT AURICLE.

At the same meeting of the Clinical Society Dr. Isambard Owen and Dr. W. J. Fenton related a case of enormous dilatation of the left auricle of the heart, the capacity of which was 30 ounces, and its greatest diameter 6¼ inches, exclusive of the appendix, which was also greatly dilated. The walls of the auricle were extremely thin but healthy in structure, and the pericardium was adherent posteriorly. The other cavities of the heart were also dilated to a more or less extent, and the ventricles were hypertrophied. The mitral orifice was much enlarged and the edges and cords somewhat contracted, but no other evidences of former endocarditis were present. One point of great interest was that the heart and pericardium occupied the entire cavity of the thorax below the level of the fourth ribs in front, the pericardium being adherent to the right parietal pleura from the spine to the anterior axillary line. As a result it happened that paracentesis was performed in the sixth space and mid-axillary line, in the hope of relieving the urgent dyspnœa from which the patient was suffering on admission. Pure blood flowed through the canula, the trocar having, as was afterwards found, penetrated the left auricle of the heart. The operation was of course at once suspended. The patient appeared none the worse for the occurrence, and survived for three and a half days, dying eventually from asphyxia due to compression of the lungs. It was extraordinary how large a proportion of the cavity of the chest was occupied by this enormous left auricle, and especially how extensively it intruded on the right side. The physical signs which led to the performance of the operation, in the presence, of course, of urgent dyspnœa for which it seemed necessary to obtain relief, were as follows:—The apex beat was in the eighth space and in the posterior axillary line; a heaving impulse was felt over the front of the chest; a systolic murmur and a sharp second sound were heard over the front of the chest, culminating at the apex; a presystolic murmur was also occasionally heard. Then there was loss of vocal fremitus, as well as of percussion resonance and stethescopic sounds, over the right base and flank. Who was to imagine that with the left side of the chest so largely occupied by heart as was here the case, this dulness was heart also? Yet so it was. It was a very rare if not unique condition, the etiology of which was by no means clear. The general opinion seemed to be that the first origin of the dilatation of the auricle had been the adhesion of the pericardium to its walls, although the active dilating force was the ventricular contraction; but it must be confessed that one has often seen a good deal of such adhesion without such results.

